# NRAS mutations in cutaneous T cell lymphoma (CTCL) sensitize tumors towards treatment with the multikinase inhibitor Sorafenib

**DOI:** 10.18632/oncotarget.17669

**Published:** 2017-05-07

**Authors:** Michael K. KieΔling, Jan P. Nicolay, Tabea Schlör, Claus-Detlev Klemke, Dorothee Süss, Peter H. Krammer, Karsten Gülow

**Affiliations:** ^1^ German Cancer Research Center, 69120 Heidelberg, Germany; ^2^ Department of Dermatology, Venerology and Allergology, University Medical Center Mannheim, Ruprecht Karls University of Heidelberg, 68167 Mannheim, Germany; ^3^ Current address: Department of Gastroenterology, University Hospital of Zürich, 8091 Zürich, Switzerland; ^4^ Current address: Department of Dermatology, Venerology and Allergology, General Hospital Karlsruhe, 76187 Karlsruhe, Germany

**Keywords:** T cell lymphoma, RAS mutation, kinase, small molecule inhibitor, targeted therapy

## Abstract

Therapy of cutaneous T cell lymphoma (CTCL) is complicated by a distinct resistance of the malignant T cells towards apoptosis that can be caused by NRAS mutations in late-stage patients. These mutations correlate with decreased overall survival, but sensitize the respective CTCL cells towards MEK-inhibition-induced apoptosis which represents a promising novel therapeutic target in CTCL. Here, we show that the multi-kinase inhibitor Sorafenib induces apoptosis in NRAS-mutated CTCL cells. CTCL cell lines and to a minor extent primary T cells from Sézary patients without NRAS mutations are also affected by Sorafenib-induced apoptosis suggesting a sensitizing role of NRAS mutations for Sorafenib-induced apoptosis. When combining Sorafenib with the established CTCL medication Vorinostat we detected an increase in cell death sensitivity in CTCL cells. The combination treatment acted synergistically in apoptosis induction in both non-mutant and mutant CTCL cells. Mechanistically, this synergistic apoptosis induction by Sorafenib and Vorinostat is based on the downregulation of the anti-apoptotic protein Mcl-1, but not of other Bcl-2 family members. Taken together, these findings suggest that Sorafenib in combination with Vorinostat represents a novel therapeutic approach for the treatment of CTCL patients.

## INTRODUCTION

Cutaneous T cell lymphoma (CTCL) is a rare malignancy of T lymphocytes homing to the skin. The malignant T cell population in CTCL is characterized by a distinct resistance towards apoptosis that causes high relapse rates and complicates therapy [1, 2]. Consequently, no curative treatment has been established for CTCL yet. Thus novel treatment options are needed for patients with relapsing or late-stage disease. In recent years, a wide variety of aberrations on the genetic, molecular and signalling level have been identified that contribute to the pathology of the disease and might represent promising novel therapeutic targets for the development of more efficient CTCL treatment options [3–6]. We have published a screen based on OncoMap technology and on Illumina PCR sequencing revealing mutations in the RAS pathway of CTCL patients [7]. Here, we found that KRAS and NRAS mutations occurred exclusively in stage IV patients indicating that mutations are associated with late-stage disease [7]. In addition, we revealed an oncogenic NRAS ^Q61K^ mutation in the CTCL cell line Hut78 that results in hyperactivation of the RAS pathway [7]. Somatic mutations in many cancers including colon carcinoma, melanoma, or pancreatic cancer are often found in BRAF, KRAS, or NRAS [8–11]. Typical mutations keep RAS in an activated form and affect glycine 12 (G12), glycine 13 (G13), or glutamine 61 (Q61) wheras BRAF is activated by valin 600 mutation [8–10].

The RAS pathway is involved in the regulation of cellular responses to environmental stimuli and plays an important role in cancer [12, 13]. In tumor cells oncogenic RAS preferentially promotes survival and proliferation [12, 13]. Thus, RAF and MEK are attractive therapeutic targets [14, 15]. BRAF mutations have gained increasing importance in recent years as targets for specific kinase inhibitors which are already in successful clinical use in oncologic therapy [15–18]. In advanced melanoma, for example, phase II and III studies showed that NRAS-mutated tumors respond to MEK inhibitors alone [7, 19–22]. Indeed, we discovered that the NRAS^Q61K^ mutation in Hut78 cells sensitized towards treatment with inhibitors of MEK kinases *in vitro* [7]. For this study we wanted to explore the multikinase inhibitor Sorafenib (Nexavar^®^, BAY 43-9006) which is already approved for clinical treatment of renal and hepatocellular carcinoma (RCC, HCC) as well as for thyroid carcinoma [23–26]. Sorafenib blocks CRAF and BRAF activity *in vitro* with an IC_50_ of 2 and 25 nM, respectively [27]. In addition, it is known that Sorafenib also targets other kinases including VEGFR-2, Flt-3, c-Kit, and PDGFRb further broadening its inhibitory action on growth of tumor cells [27, 28]. Unfortunately, Sorafenib failed to be a specific inhibitor for mutant BRAF melanomas. This was a demotivating result [29], however, Sorafenib shows a certain broad and maybe unspecific effect on blocking the RAS signalling pathway [27]. Interestingly, a recent pilot study found clinical activity of Sorafenib in patients with T cell lymphoma with 44% partial and 11% complete responses. However, these responses were of short duration between 1 and 2.8 months [30]. Thus, we wanted to investigate Sorafenib in CTCL and wondered whether this initial therapeutic effect could be further enhanced by combination therapies. Since Sorafenib and Vorinostat target multiple overlapping pathways implicated in tumor cell survival, it is possible that a combination of both agents might be more effective than either agent alone [31–33].

Here we show that Sorafenib blocks cell growth in CTCL cell lines but preferentially in Hut78 which harbours an oncogenic NRAS ^Q61K^ mutation. In concurrence with the previous finding Sorafenib induced apoptosis was most prominent in Hut78 cells. A specific inhibitor for mutated BRAF ^V600E^, PLX4720, had no effect on survival of CTCL cell lines. Further, current treatment with Sorafenib and the HDAC inhibitor Vorinostat induces cell death in a synergistic manner in CTCL cell lines and in primary tumor cells from Sézary patients. Sorafenib together with Vorinostat caused a significant downregulation of the anti-apoptotic protein Mcl-1. In accordance, overexpression of Mcl-1 blocked apoptosis induced by Sorafenib and Vorinostat. Thus, Sorafenib in combination with Vorinostat may be used as a drug in non-mutant and CTCL patients displaying a RAS mutation.

## RESULTS

### The RAF kinase inhibitor Sorafenib blocks MEK-ERK signaling after PMA stimulation and inhibits cell growth in CTCL cell lines

**Figure 1:**
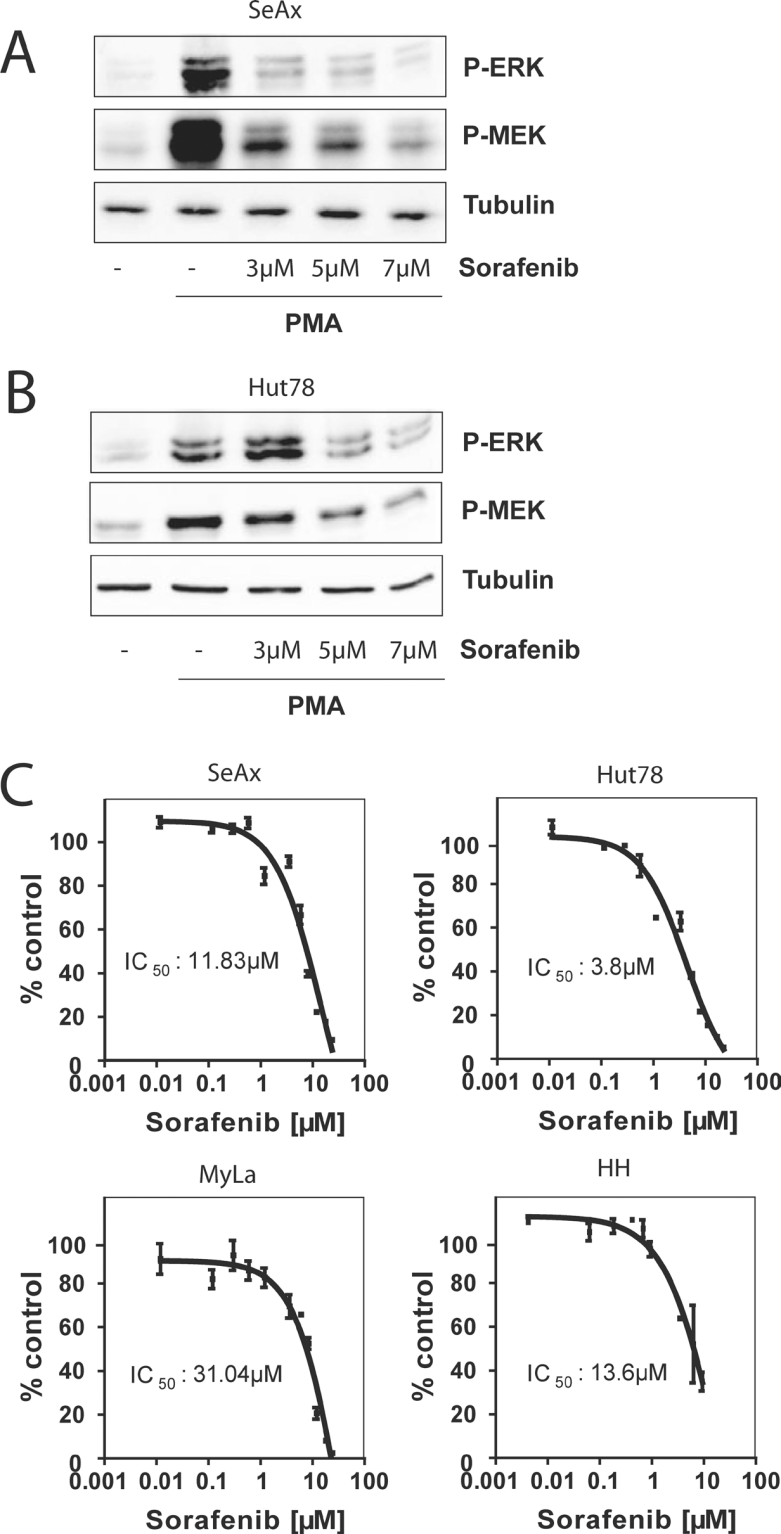
Sorafenib blocks RAS signaling and inhibits cell growth Cells were left untreated, stimulated with PMA, or pre-treated with 3 μM, 5 μM, and 7μ M of Sorafenib for 30 min and then stimulated with PMA. Then, cells were lysed and the phosphorylation level of ERK and MEK was assessed by Western blot with specific anti-phospho-ERK and with specific anti-phospho-MEK antibodies. Equal loading was verified by α-tubulin. (**A**) Representative Western blot of SeAx cells. (**B**) Representative Western blot of Hut78 cells. (**C**) CTCL cell lines were incubated with indicated concentrations of the pan-RAF inhibitor Sorafenib for 72 hours. Cell growth was measured by Cell Titer Glo according to manufactor´s instructions. The IC_50_ value represents the Sorafenib concentration that inhibits 50% cell growth compared to DMSO treated control cells. The IC_50_ was calculated by GraphPad Prism software (San Diego, CA).

RAS mutations occur in about 11% of CTCL patients at advanced disease stage IV [7]. This prompted us to ask whether RAF inhibitors could be of relevance for the treatment of patients bearing a RAS mutation. To evaluate the inhibitory effect of Sorafenib on the RAS-RAF pathway we analyzed phosphorylation levels of the MEK-ERK cascade by Western blot. In phorbol 12-myristate 13-acetate (PMA) stimulated Hut78 and SeAx cells Sorafenib inhibited MEK and ERK phosphorylation at concentrations between 3 μM and 7 μM (Figure 1A, 1B). This finding suggests that Sorafenib is able to execute its inhibitory function on RAS-RAF-MEK-ERK signaling. In addition, we checked for the inhibitory effect of Sorafenib on RAS-RAF signaling by comparing differences in cell growth of CTCL cell lines using Cell Titer Glow. We observed that Hut78 which harbours a NRAS mutation has a significantly lower IC_50_ (3.8 μM) compared to SeAx or MyLa cells (11.8 μM and 31.04 μM, respectively). This data shows that RAS mutations sensitize towards treatment with multikinase inhibitor Sorafenbi (Figure 1C).

### Oncogenic NRAS^Q61K^ is critical for survival of Hut78 cells

**Figure 2:**
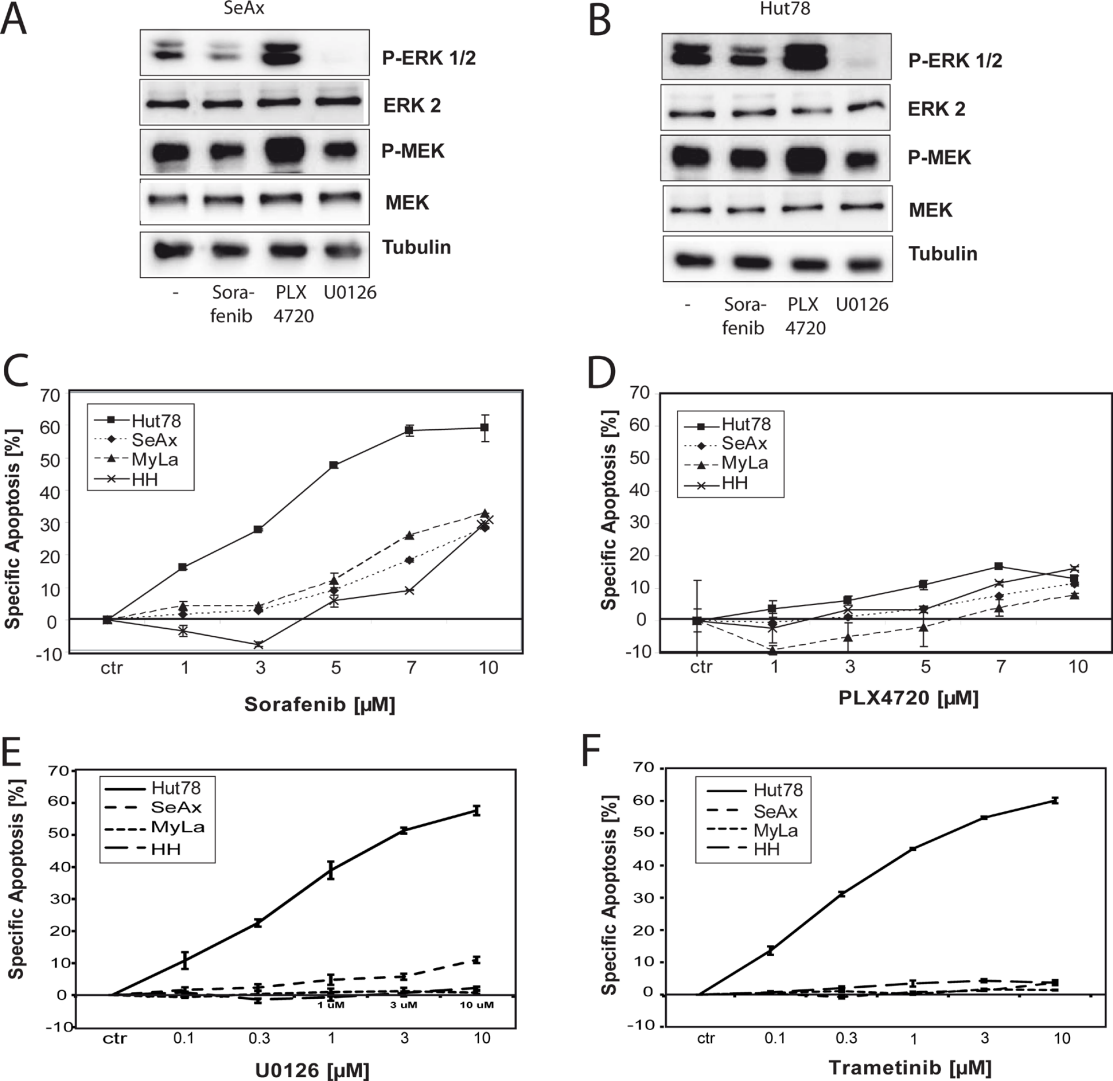
NRAS^Q61K^ sensitizes towards treatment with Sorafenib Cells were left untreated or treated with 10 μM of Sorafenib, 3 μM of PLX4720, or 1μM of U0126 for 4 hours. Then, cells were lysed and the basal phosphorylation level of ERK and MEK was assessed by Western blot with specific anti-phospho-ERK and with specific anti-phospho-MEK antibodies. Equal loading was verified by α-tubulin, total ERK and total Mek. (**A**) Representative Western blot of SeAx cells. (**B**) Representative Western blot of Hut78 cells. (**C**–**F**) All four CTCL cell lines were incubated with indicated concentrations of the used inhibitors for 48 hours. Then, apoptosis was determined and specific apoptosis was calculated according to the description in materials and methods. Data shown is representative for at least three independent experiments. (C) Cells were treated with the pan-RAF inhibitor Sorafenib. (D) Cells were incubated with indicated concentrations of the BRAF specific inhibitor PLX4720. (E, F) Similar to (C), however, all four CTCL cell lines were incubated with indicated concentrations of the MEK inhibitor U0126 (E) or Trametinib (F) for 48 h hours. Data shown is representative for two independent experiments.

In order to evaluate different types of inhibitors of the RAS pathway on CTCL cells, we checked the effect of Sorafenib, PLX4720 and U0126 on basal ERK and MEK phosphorylation in SeAx and Hut78 cells. SeAx cells showed lower basal activation of the MEK/ERK pathway compared to Hut78 cells harbouring the RAS mutation [7]. However, Sorafenib blocked MEK and ERK phosphorylation in both cell lines, Hut78 and SeAx cells, respectively (Figure 2A, 2B). As reported, the specific BRAF^V600E^ inhibitor PLX4720 increased both, MEK and ERK phosphorylation in SeAx and Hut78 cells since both cell lines express wild type BRAF [34, 35]. The MEK inhibitor U0126 showed the strongest inhibitory effect for both cell lines (Figure 2A, 2B). In order to investigate whether NRAS^Q61K^ also sensitizes towards RAF inhibition in CTCL, we treated all four cell lines with Sorafenib and measured cell death after 48 hours. Sorafenib induced apoptosis in all four CTLC cell lines (up to 60% in Hut78 and up to 30% in SeAx, HH, and Myla; Figure 2C). In addition, Sorafenib also induced apoptosis of lower concentrations in primary T cells from Sézary patients (treatment with 3–7 μM Sorafenib induced up to 40% cell death) compared to healthy controls (3–7 μM induced less than 20% apoptosis; Supplementary Figure 1). This indicates that besides its effect in mutant tumor cells Sorafenib also causes cell death in non-mutant tumor cells. However, as expected, the most prominent induction of apoptosis was observed in the RAS mutant cell line Hut78 (up to 60%) (Figure 2C). Of note, apoptosis induction occurred at low molecular concentrations in the range of 1 μM to 3 μM which have been shown to be clinically relevant in plasma. Here, specific apoptosis of about 30% in hepato cellular carcinoma (HCC) cell lines was reached at concentrations of 10 μM Sorafenib at 48 hours [36]. We observed apoptosis already at lower Sorafenib concentrations indicating that a clinically relevant effect in treating CTCL can be expected. Recent studies revealed that BRAF inhibitors exclusively block tumor growth in tumors harbouring BRAF^V600E^ mutations since CRAF can over-compensate for BRAF inhibition [34, 35]. We could confirm this data by using the specific BRAF inhibitor PLX4720. PLX4720 rather induced than blocked ERK phosphorylation (Figure 2A, 2B). Consequently, PLX4720 treatment led to minor induction of apoptosis (not more than 20% cell death after 48 hours treatment for all four CTCL cell lines; Figure 2D). Interestingly, we observed that the MEK inhibitor U0126 caused a strong inhibition of basal ERK phosphorylation (Figure 2A, 2B) and induced apoptosis specifically in Hut78 cells (more than 50% upon 48 hours of treatment) whereas the other cell lines were not/less effected (Figure 2E). The clinical approved Mek inhibitor Trametinib basically displayed the same result (more than 60% cell death in Hut78 cells and only minor effects in non-mutaned cell lines; Figure 2F). Taken together, these results show that NRAS mutations confer sensitivity towards inhibitors of the RAS-RAF-MEK-ERK pathway.

### Sorafenib and Vorinostat synergistically induce apoptosis in CTCL cell lines

**Figure 3:**
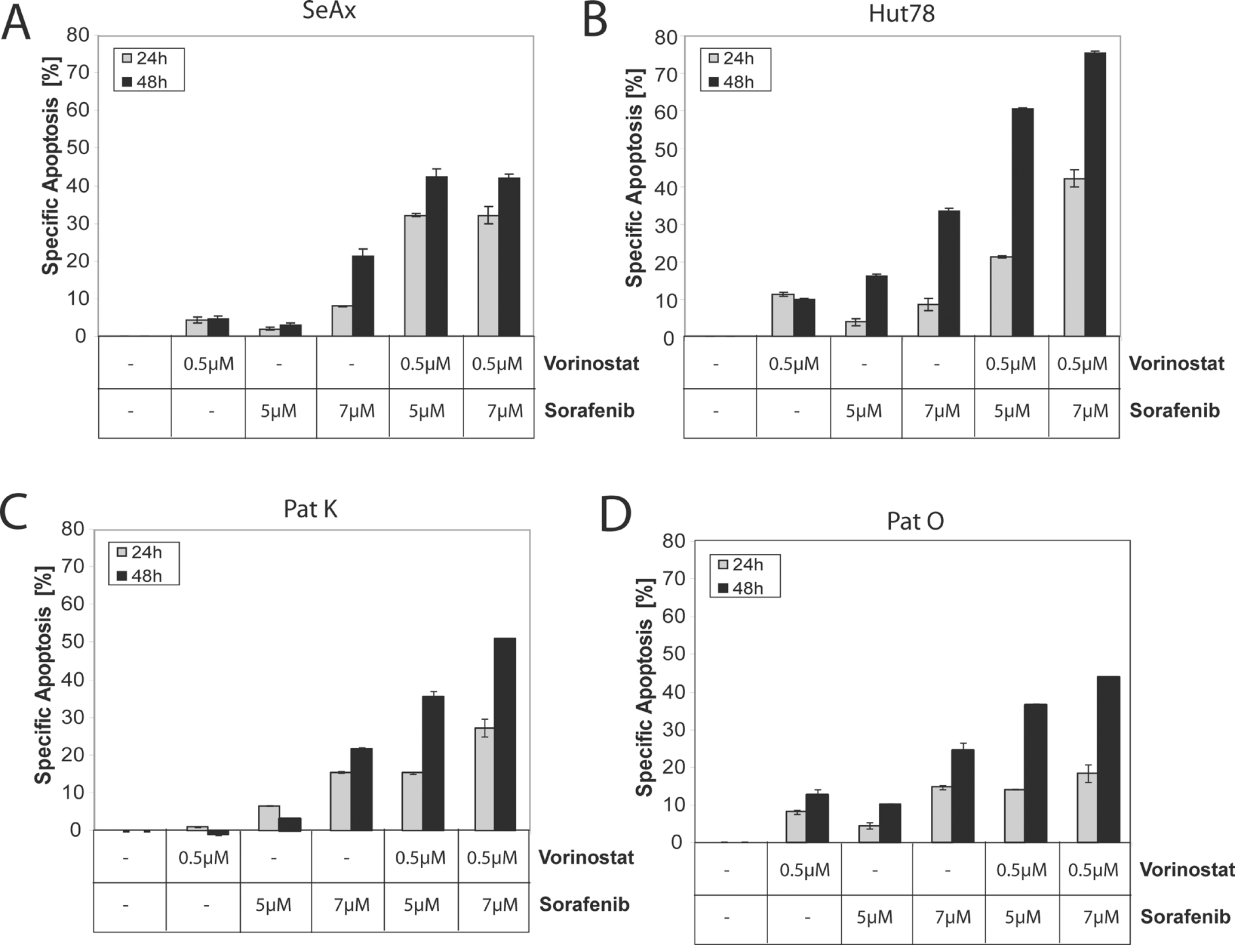
Concurrent treatment with Sorafenib and Vorinostat synergistically induce apoptosis in non-mutant and in mutant cells Cells were left untreated, treated with either Vorinostat or Sorafenib alone, or treated with different combinations of Vorinostat and Sorafenib for 24 and 48 hours. Then, apoptosis was determined and specific apoptosis was calculated according to the description in materials and methods. (**A**) SeAx were used. (**B**) Hut78 cells were used. (**C**, **D**) Same as (A, B), but CD4^+^ T cells isolated from two different patients were used.

Vorinostat (Zolinza^®^), a histone deacetylase inhibitor, was approved for the treatment of CTCL after a phase IIb clinical trial showing clinical safety and efficiency [37]. Vorinostat is described to induce histone acetylation, cell cycle arrest, and apoptosis in CTCL cell lines [38]. However, the overall response rate (ORR) of CTCL patients towards monotherapy with Vorinostat (30%) remains poor [37]. In addition, Sorafenib shows early promising activity as monotherapy [30]. Interestingly, several studies proposed a synergistic action of Vorinostat and Sorafenib to induce apoptosis in tumor cells of different malignancies [31, 32, 39]. To investigate a putative synergistic effect of Vorinostat and Sorafenib, non-mutant and mutant CTCL cell lines were incubated with different combinations of both drugs. Interestingly, concurrent treatment of Sorafenib and Vorinostat synergistically induced apoptosis in the non-mutant cell line SeAx that previously showed only modest sensitivity to Sorafenib treatment alone (Figure 3A). Synergism was calculated according to the test of Jonkheere [40, 41]. However, synergistic apoptosis levels after 24 and 48 hours of treatment were lower in the non-mutant SeAx cell line compared to the mutant Hut78 cell line (Figure 3A, 3B). Concurrent treatment with low concentrations of both inhibitors, further enhanced Sorafenib-induced apoptosis in the mutant cell line Hut78 (Figure 3B). Again this was a synergistic effect as calculated with the Jonkheere method. To assess whether synergistic action can be observed in primary cells, we isolated Sézary cells and cotreated them with both inhibitors. Sorafenib and Vorinostat induced apoptosis in a synergistic manner in Sézary cells from two different patients (Figure 3C, 3D). Both patients showed wild-type status for NRAS, KRAS, HRAS, or BRAF. Co-incubation of T cells from healthy donors with Sorafenib and Vorinostat showed no cooperative effect of both drugs. To the contrary, not even an additive effect could be observed indicating that these drugs need malignant cell transformation for demonstrating synergism even in absence of RAS mutations (Supplementary Figure 2). In conclusion, Sorafenib increases the toxic activity of Vorinostat in both, non-mutant and mutant tumor cells.

### Sorafenib and Vorinostat synergistically downregulate the anti-apoptotic protein Mcl-1

**Figure 4:**
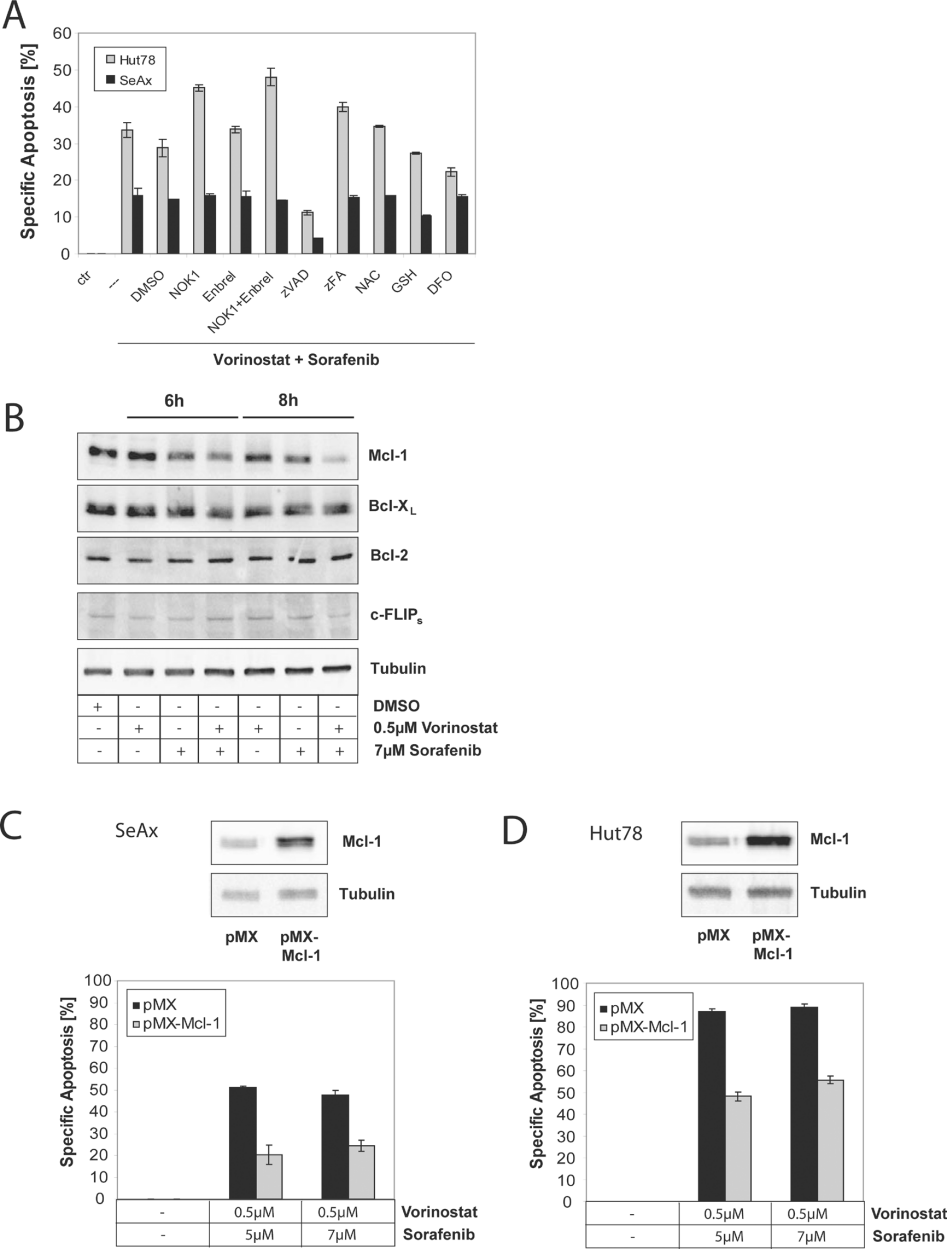
Sorafenib and Vorinostat induce apoptosis synergistically *via* down-regulation of Mcl-1 (**A**) Hut78 or SeAx cells treated with either 0.5 μM Vorinostat and 5 μM Sorafenib alone, or co-treated with 1% DMSO, 1% NOK 1, 25 μM Enbrel^®^, 20 μM zVAD, 50 μM zFA, 20 mM N-acetyl-cysteine (NAC), 0.5 mM glutathione monoethyl ester (GSH), or 20 μM desferrioxamine (DFO) for 24 and 48 hours. Then, apoptosis was determined by flow cytometry and specific apoptosis was calculated according to the description in materials and methods. (**B**) SeAx cells were left untreated, treated with either Vorinostat or Sorafenib alone, or treated with different combinations of Vorinostat and Sorafenib for 6 and 9 hours. Then, cells were lysed and lysates subjected to Western blot. Expression of Bcl-2 family members was assessed by specific antibodies for Mcl-1, Bcl-2, and Bcl-xL, or anti-apoptotic protein c-FLIPs. Equal loading was verified by anti-tubulin antibodies. (**C**) SeAx cells were transfected with empty retroviral vector pMX or pMX encoding for Mcl-1. Cells were sorted for GFP co-expression. Then, cells were stimulated with different combinations of Vorinostat and Sorafenib for 48 hours. Next, apoptosis was determined and specific apoptosis was calculated according to the description in materials and methods. Inserts show overexpression of Mcl-1 in pMX-Mcl-1 vector transfected cell compared to pMX control transfected cells. (**D**) Same as in (D), but Hut78 cells were used.

Various mechanisms were suggested to explain the synergistic action of Sorafenib and Vorinostat. Among them, CD95 upregulation, c-FLIP suppression, or alteration in Mcl-1 expression were discussed [31, 32]. We did not observe any involvement of the CD95/CD95L system in CD95L blocking experiments using a neutralizing anti-CD95L antibody (NOKI); TNF signaling was ruled out by inhibition using Etanercept (Enbrel^®^) (TNF-R-Fc); and a major contribution by reactive oxygen species (ROS) was excluded by experiments using antioxidants such as N-acetyl-cysteine (NAC), glutathione monoethyl ester (GSH), or the iron chelator desferrioxamine (DFO) (Figure 4A). However, we found that apoptosis induced by Sorafenib and Vorinostat is dependent on caspases as verified by the pan-caspase inhibitor zVAD (Figure 4A). To further investigate the mode of action of concurrent Sorafenib and Vorinostat treatment, we studies expression levels of Bcl-2 family members. Here, we found that both drugs together result in significant downregulation of Mcl-1 but not of other members of the Bcl-2 family or c-FLIP (Figure 4B). In addition, we overexpressed Mcl-1 using a retroviral overexpression vector pMX and incubated either SeAx cells displaying wilde-type RAS expression or Hut78 cells with different combinations of Sorafenib and Vorinostat. Overexpression of Mcl-1 blocked apoptosis up to 50% in both cell lines demonstrating an important role of Mcl-1 as a RAS/RAF target gene in mediating survival and apoptosis resistance in mutant and non-mutant CTCL cells (Figure 4C, 4D).

## DISCUSSION

We found that activating NRAS mutations in CTCL cell lines and patient cells sensitize towards treatment with the multikinase inhibitor Sorafenib. This confirms previous data showing that NRAS^Q61R^ mutations sensitize different benign and malignant cell types towards MEK inhibition or that NRAS^G13A^ mutations confer resistance towards apoptosis [7, 42–45]. Previous data shows that aberrant activation of the MAPK pathway including NRAS not only derives from NRAS mutations. This pathway interacts with other signaling pathways that have been shown to be altered in CTCL cells including NFκB, TCR and PLCγ1 signaling, respectively [6]. Thus, these pathways can induce MAPK activation even in non-mutant cells. In addition, it has been shown that deregulated or deleted transcription factors like E2A can lead to an indirect activation of the RAS pathway *via* downregulation of inhibitory factors like RasSF4 [46]. These findings on aberrant MAPK activation in CTCL cells suggest Sorafenib as a promising treatment option for late-stage CTCL patients with RAS mutations. Of note, apoptosis induction by Sorafenib occurred at low molecular concentrations in the range of 1 μM to 3 μM which has been shown to be clinically relevant in plasma. Specific apoptosis of about 30% in HCC cell lines was reached at concentrations of 10 μM Sorafenib after 48 hours of treatment [36]. We observed apoptosis already at lower concentrations of Sorafenib. Since Sorafenib does not exclusively inhibit BRAF and CRAF but also blocks several other pathways these could be involved in effectiveness against CTCL cells. Still, inhibition of MEK – the main RAS/RAF target - by a specific inhibitor also induced cell death preferentially in Hut78 cells (Figure 2). Yet, the MEK inhibitor caused a stronger downregulation of basal ERK signaling than Sorafenib (Figure 2) suggesting that Sorafenib works also *via* different pathways and that Sorafenib is not as efficient in blocking ERK signaling as MEK inhibitors. This pathway may include the known target genes EGFR, PDGFR, c-Kit and Flt-3. In addition, Sorafenib inhibits the RAS pathway and, thus, sensitizes Hut78 cells. It was reported that Sorafenib is not a specific BRAF^V600E^ inhibitor (14). The specific BRAF^V600E^ mutation is important for putative treatment of late-stage CTCL patients with confirmed NRAS or KRAS mutations. The effect of Sorafenib or of MEK inhibitors such as AZD6244 or PD0325901 have to be evaluated on primary patient cells to choose optimal treatment conditions. We found that four out of 90 patients harboured mutations for NRAS and KRAS [7]. Unfortunately, three of these four patients died due to progression of the disease, whereas one patient dropped out during follow-up preventing further investigations of primary CTCL cells in our study.

Previously, we have observed that knock-down of NRAS by siRNA induces apoptosis in Hut78 cells only but not in non-mutated RAS cell lines showing that Hut78 depends on hyperactive RAS-RAF signaling [7]. This specific dependence of RAS-RAF-MEK-ERK signaling was further corroborated by the results obtained by using the MEK inhibitor U0126 which caused apoptosis in Hut78 cells only. Two publications suggest that a hyperactivated RAS pathway results in downregulation of the anti-apoptotic Bcl-2 family member Bim, thereby preventing apoptosis [44, 47]. However, we could not observe an effect on Bim induced by inhibitor treatment suggesting that BIM is not involved in this process (data not shown). In addition, inhibition of the RAF-RAS pathway by Sorafenib was shown to cause downregulation of the anti-apoptotic protein Mcl-1 in hepatocytes [27, 36]. This was now confirmed for the CTCL cell line Hut78 where Sorafenib alone led to diminished expression of Mcl-1 (Figure 4). Vorinostat is approved for the treatment of CTCL in several countries, although it has limited therapeutic success as a monotherapy [37, 48]. Nevertheless, it was shown in pre-clinical studies that Vorinostat together with Sorafenib can act synergistically in other tumor entities [31–33]. Although the mechanism for this pharmacologic synergism is not completely elucidated yet, there are several findings that contribute to its explanation. It has been found recently, that RAS mutations can upregulate HDACs and, thus, cause resistance towards Vorinostat treatment [39]. In contrast, Sorafenib has been shown to induce a downregulation of HDACs and, thus, to potentiate histone acetylation, so that it thereby also potentiates the epigenetic effect of Vorinostat further suggesting the use of the combination of Sorafenib and Vorinostat [49]. Indeed, concurrent treatment with Sorafenib and Vorinostat induced apoptosis in a synergistic manner in the mutant CTCL cell line Hut78 (Figure 3). The synergistic action was also observed for T cells of CTCL patients not bearing a RAS mutation indicating that the effect of Sorafenib can be enhanced in non-mutant tumor cells as well (Figure 3). Interestingly, T cells of healthy donors showed no synergistic cell death induction upon Sorafenib and Vorinostat cotreatment suggesting that malignant transformation and apoptosis resistance of T cells is necessary for the synergistic therapeutic effect of both drugs. With respect to the underlying mechanism of this synergistic effect, we detected that Sorafenib and Vorinostat synergize in downregulation of the anti-apoptotic Bcl-2 family member Mcl-1 (Figure 4). Mcl-1 downregulation was identified as a major part of the synergistic mechanism. Nevertheless, the histone acetylation-promoting effect of Sorafenib is likely to contribute to the synergistic effect and can, therefore, explain the restriction of the synergism to malignant T cells. Thus, Sorafenib in combination with Vorinostat may serve as a new treatment strategy for RAS mutant or RAS non-mutant CTCL patients. This is of particular interest, as first clinical data of a phase 1 pilot study showed encouraging results of Sorafenib treatment in CTCL patients with a complete remission in 11% of patients [30]. Although the results of this study are limited by the small number of patients it confirms our findings and further promotes Sorafenib as a possible future CTCL treatment option, especially in combination with other CTCL medication that targets different pathways, especially HDAC inhibitors like Vorinostat.

## MATERIALS AND METHODS

### Chemicals

Sorafenib was provided by Bayer Schering Pharma (Bayer Vital GmbH). PLX4720 was purchased by Symansis, U0126 was purchased from Sigma-Aldrich, Trametinib was obtained from Target molecule Corp, and Vorinostat (SAHA) was purchased from Selleck. Deferrioxamine Mesylate (DFO) and Glutathione-monoethyl-ester (GSH) were obtained from Merck., N-Acetyl-L-cysteine (NAC) was purchased from Sigma-Aldrich. Pancaspase inhibitor zVAD and cathepsin inhibitor Z-FA-fmk were purchased from R&D Systems. Etanercept (Enbrel^®^) was kindly provided by Dr. Walczak, Imperial College London.

### Cell culture

The CTCL cell lines SeAx [50], Hut78 [51], MyLa [52], and HH [53] were cultured in Roswell Park Memorial Institute (RPMI) medium supplemented with 10% FCS and 1 mM L-Glutamine. SeAx, MyLa, and HH cells were obtained from Stefan Eichmüller, German Cancer Research Center, Heidelberg, Germany. Hut78 cells were purchased from ATCC. SeAx, Hut78, MyLa, and HH cells were tested by the cell contamination control of German Cancer Research Center, Heidelberg, Germany using a multiplex cell contamination test [54].

### Patients

Four patients with Sézary syndrome (CTCL stage IV) diagnosed according to World Health Organization-European Organization of Research and Treatment of Cancer classification of CTCL and criteria of the international society of cutaneous lymphomas were included in the study (see Supplementary Table 1). Informed consent was obtained from all subjects before inclusion. The study was conducted according to ethical guidelines at our institution and the Helsinki Declaration and was approved by the ethics committee II of the University of Heidelberg.

### Western blot analysis

1 × 10^6^ CTCL cells were lysed for 10 min in ice-cold RIPA lysis buffer (50 mM Tris-HCl, pH 8.0, 120 mM NaCl, 1% NP-40, 0.5% Na-Desoxycholat, 0.1% SDS, 2 mM EDTA, 25 mM NaF, 0.2 mM NaVO_4_, 1 mM DTT, and complete protease inhibitor cocktail from Roche). Lysates were separated by SDS-PAGE and proteins were blotted onto a nitrocellulose membrane (Amersham Biosciences) followed by blocking with 5% BSA in PBS/Tween (0.05% Tween-20 in PBS). The following antibodies were used: anti-phospho-ERK (P-p44/p42 (Tyr202/204); Cell Signalling), anti-ERK2 (C-14; Santa Cruz), anti-phospho-MEK1/2 ((Ser217/212); Cell Signalling), anti-MEK1/2 (Cell Signalling), anti-Mcl-1 (S-19; Santa Cruz), anti-tubulin (Sigma-Aldrich).

### Lymphocyte separation

Human peripheral blood leukocytes were purified as described previously [55]. Then, T cells were sorted by CD4^+^ surface staining with the CD4^+^ T Cell Isolation Kit II according to the manufacture's instruction (Miltenyi Biotec). The study was conducted according to the ethical guidelines of the German Cancer Research Center and the Helsinki Declaration, and it was approved by the ethics committee II of the Ruprecht-Karls-University of Heidelberg, Germany. Primary T cells were cultured in RPMI 1640 supplemented with 10% FCS.

### Cell death assays

For cell death induction, CTCL cells were stimulated with the indicated concentrations of inhibitors. Cell death was assessed by forward-to-side-scatter (FSC/SSC) profile [56]. Specific cell death was calculated using the following equation: specific cell death % = (% experimental cell death - % spontaneous cell death) / (100%–% spontaneous cell death) × 100 [55].

## SUPPLEMENTARY FIGURES AND TABLE



## References

[1] Jawed SI, Myskowski PL, Horwitz S, Moskowitz A, Querfeld C (2014). Primary cutaneous T-cell lymphoma (mycosis fungoides and Sezary syndrome): part II. Prognosis, management, and future directions. J Am Acad Dermatol.

[2] Whittaker S, Hoppe R, Prince HM (2016). How I treat mycosis fungoides and Sezary syndrome. Blood.

[3] Ungewickell A, Bhaduri A, Rios E, Reuter J, Lee CS, Mah A, Zehnder A, Ohgami R, Kulkarni S, Armstrong R, Weng WK, Gratzinger D, Tavallaee M (2015). Genomic analysis of mycosis fungoides and Sezary syndrome identifies recurrent alterations in TNFR2. Nat Genet.

[4] da Silva Almeida AC, Abate F, Khiabanian H, Martinez-Escala E, Guitart J, Tensen CP, Vermeer MH, Rabadan R, Ferrando A, Palomero T (2015). The mutational landscape of cutaneous T cell lymphoma and Sezary syndrome. Nat Genet.

[5] Wang L, Ni X, Covington KR, Yang BY, Shiu J, Zhang X, Xi L, Meng Q, Langridge T, Drummond J, Donehower LA, Doddapaneni H, Muzny DM (2015). Genomic profiling of Sezary syndrome identifies alterations of key T cell signaling and differentiation genes. Nat Genet.

[6] Nicolay JP, Felcht M, Schledzewski K, Goerdt S, Geraud C (2016). Sezary syndrome: old enigmas, new targets. J Dtsch Dermatol Ges.

[7] Kiessling MK, Oberholzer PA, Mondal C, Karpova MB, Zipser MC, Lin WM, Girardi M, Macconaill LE, Kehoe SM, Hatton C, French LE, Garraway LA, Polier G (2011). High-throughput mutation profiling of CTCL samples reveals KRAS and NRAS mutations sensitizing tumors toward inhibition of the RAS/RAF/MEK signaling cascade. Blood.

[8] (2010). BRAF mutations in cancer. http://wwwsangeracuk/perl/genetics/CGP/cosmic?action=bygene&ln=BRAF&start=&end=&coords=AA%3AAA.

[9] (2010). KRAS mutations in cancer. http://wwwsangeracuk/perl/genetics/CGP/cosmic?action=bygene&ln=KRAS&start=&end=&coords=AA%3AAA.

[10] (2010). NRAS mutations in cancer. http://wwwsangeracuk/perl/genetics/CGP/cosmic?action=bygene&ln=NRAS.

[11] Hobbs GA, Der CJ, Rossman KL (2016). RAS isoforms and mutations in cancer at a glance. Journal of cell science.

[12] Kan Z, Jaiswal BS, Stinson J, Janakiraman V, Bhatt D, Stern HM, Yue P, Haverty PM, Bourgon R, Zheng J, Moorhead M, Chaudhuri S, Tomsho LP (2010). Diverse somatic mutation patterns and pathway alterations in human cancers. Nature.

[13] Burotto M, Chiou VL, Lee JM, Kohn EC (2014). The MAPK pathway across different malignancies: a new perspective. Cancer.

[14] Braun BS, Shannon K (2008). Targeting Ras in myeloid leukemias. Clin Cancer Res.

[15] Richman J, Martin-Liberal J, Diem S, Larkin J (2015). BRAF and MEK inhibition for the treatment of advanced BRAF mutant melanoma. Expert Opin Pharmacother.

[16] Chapman PB, Hauschild A, Robert C, Haanen JB, Ascierto P, Larkin J, Dummer R, Garbe C, Testori A, Maio M, Hogg D, Lorigan P, Lebbe C (2011). Improved survival with vemurafenib in melanoma with BRAF V600E mutation. N Engl J Med.

[17] Hauschild A, Grob JJ, Demidov LV, Jouary T, Gutzmer R, Millward M, Rutkowski P, Blank CU, Miller WH, Kaempgen E, Martín-Algarra S, Karaszewska B, Mauch C (2012). Dabrafenib in BRAF-mutated metastatic melanoma: a multicentre, open-label, phase 3 randomised controlled trial. Lancet.

[18] Long GV, Stroyakovskiy D, Gogas H, Levchenko E, de Braud F, Larkin J, Garbe C, Jouary T, Hauschild A, Grob JJ, Chiarion-Sileni V, Lebbe C, Mandala M (2015). Dabrafenib and trametinib versus dabrafenib and placebo for Val600 BRAF-mutant melanoma: a multicentre, double-blind, phase 3 randomised controlled trial. Lancet.

[19] Holkova B, Zingone A, Kmieciak M, Bose P, Badros AZ, Voorhees PM, Baz R, Korde N, Lin HY, Chen JQ, Herrmann M, Xi L, Raffeld M (2016). A Phase II Trial of AZD6244 (Selumetinib, ARRY-142886), an Oral MEK1/2 Inhibitor, in Relapsed/Refractory Multiple Myeloma. Clin Cancer Res.

[20] Dummer R, Robert C, Chapman BP, Kirkwood JM (2008). AZD6244 (ARRY-142886) vs temozolomide (TMZ) in patients (pts) with advanced melanoma: an open-label, randomized, multicenter, phase II study. J Clin Oncol.

[21] Bennouna J, Lang I, Valladares-Ayerbes M, Boer K, Adenis A, Escudero P, Kim TY, Pover GM, Morris CD, Douillard JY (2011). A Phase II, open-label, randomised study to assess the efficacy and safety of the MEK1/2 inhibitor AZD6244 (ARRY-142886) versus capecitabine monotherapy in patients with colorectal cancer who have failed one or two prior chemotherapeutic regimens. Invest New Drugs.

[22] Banerji U, Camidge DR, Verheul HM, Agarwal R, Sarker D, Kaye SB, Desar IM, Timmer-Bonte JN, Eckhardt SG, Lewis KD, Brown KH, Cantarini MV, Morris C (2010). The first-in-human study of the hydrogen sulfate (Hyd-sulfate) capsule of the MEK1/2 inhibitor AZD6244 (ARRY-142886): a phase I open-label multicenter trial in patients with advanced cancer. Clin Cancer Res.

[23] Escudier B, Eisen T, Stadler WM, Szczylik C, Oudard S, Staehler M, Negrier S, Chevreau C, Desai AA, Rolland F, Demkow T, Hutson TE, Gore M (2009). Sorafenib for treatment of renal cell carcinoma: Final efficacy and safety results of the phase III treatment approaches in renal cancer global evaluation trial. J Clin Oncol.

[24] Llovet JM, Ricci S, Mazzaferro V, Hilgard P, Gane E, Blanc JF, de Oliveira AC, Santoro A, Raoul JL, Forner A, Schwartz M, Porta C, Zeuzem S (2008). Sorafenib in advanced hepatocellular carcinoma. N Engl J Med.

[25] de Castroneves LA, Negrão MV, de Freitas RM, Papadia C, Lima JV, Fukushima JT, Simão EF, Kulcsar MA, Tavares MR, Jorge AA, de Castro G, Hoff PM, Hoff AO (2016). Sorafenib for the Treatment of Progressive Metastatic Medullary Thyroid Cancer: Efficacy and Safety Analysis. Thyroid.

[26] Shen YC, Lin ZZ, Hsu CH, Hsu C, Shao YY, Cheng AL (2013). Clinical trials in hepatocellular carcinoma: an update. Liver Cancer.

[27] Wilhelm SM, Carter C, Tang L, Wilkie D, McNabola A, Rong H, Chen C, Zhang X, Vincent P, McHugh M, Cao Y, Shujath J, Gawlak S (2004). BAY 43–9006 exhibits broad spectrum oral antitumor activity and targets the RAF/MEK/ERK pathway and receptor tyrosine kinases involved in tumor progression and angiogenesis. Cancer Res.

[28] Ramakrishnan V, Timm M, Haug JL, Kimlinger TK, Halling T, Wellik LE, Witzig TE, Rajkumar SV, Adjei AA, Kumar S (2012). Sorafenib, a multikinase inhibitor, is effective in vitro against non-Hodgkin lymphoma and synergizes with the mTOR inhibitor rapamycin. Am J Hematol.

[29] Eisen T, Ahmad T, Flaherty KT, Gore M, Kaye S, Marais R, Gibbens I, Hackett S, James M, Schuchter LM, Nathanson KL, Xia C, Simantov R (2006). Sorafenib in advanced melanoma: a Phase II randomised discontinuation trial analysis. Br J Cancer.

[30] Gibson JF, Foss F, Cooper D, Seropian S, Irizarry D, Barbarotta L, Lansigan F (2014). Pilot study of sorafenib in relapsed or refractory peripheral and cutaneous T-cell lymphoma. Br J Haematol.

[31] Walker T, Mitchell C, Park MA, Yacoub A, Graf M, Rahmani M, Houghton PJ, Voelkel-Johnson C, Grant S, Dent P (2009). Sorafenib and vorinostat kill colon cancer cells by CD95-dependent and -independent mechanisms. Mol Pharmacol.

[32] Zhang G, Park MA, Mitchell C, Hamed H, Rahmani M, Martin AP, Curiel DT, Yacoub A, Graf M, Lee R, Roberts JD, Fisher PB, Grant S (2008). Vorinostat and sorafenib synergistically kill tumor cells via FLIP suppression and CD95 activation. Clin Cancer Res.

[33] Park MA, Zhang G, Martin AP, Hamed H, Mitchell C, Hylemon PB, Graf M, Rahmani M, Ryan K, Liu X, Spiegel S, Norris J, Fisher PB (2008). Vorinostat and sorafenib increase ER stress, autophagy and apoptosis via ceramide-dependent CD95 and PERK activation. Cancer Biol Ther.

[34] Hatzivassiliou G, Song K, Yen I, Brandhuber BJ, Anderson DJ, Alvarado R, Ludlam MJ, Stokoe D, Gloor SL, Vigers G, Morales T, Aliagas I, Liu B (2010). RAF inhibitors prime wild-type RAF to activate the MAPK pathway and enhance growth. Nature.

[35] Heidorn SJ, Milagre C, Whittaker S, Nourry A, Niculescu-Duvas I, Dhomen N, Hussain J, Reis-Filho JS, Springer CJ, Pritchard C, Marais R (2010). Kinase-dead BRAF and oncogenic RAS cooperate to drive tumor progression through CRAF. Cell.

[36] Liu L, Cao Y, Chen C, Zhang X, McNabola A, Wilkie D, Wilhelm S, Lynch M, Carter C (2006). Sorafenib blocks the RAF/MEK/ERK pathway, inhibits tumor angiogenesis, and induces tumor cell apoptosis in hepatocellular carcinoma model PLC/PRF/5. Cancer Res.

[37] Olsen EA, Kim YH, Kuzel TM, Pacheco TR, Foss FM, Parker S, Frankel SR, Chen C, Ricker JL, Arduino JM, Duvic M (2007). Phase IIb multicenter trial of vorinostat in patients with persistent, progressive, or treatment refractory cutaneous T-cell lymphoma. J Clin Oncol.

[38] Duvic M, Vu J (2007). Vorinostat in cutaneous T-cell lymphoma. Drugs Today (Barc).

[39] Wang Q, Tan R, Zhu X, Zhang Y, Tan Z, Su B, Li Y (2016). Oncogenic K-ras confers SAHA resistance by up-regulating HDAC6 and c-myc expression. Oncotarget.

[40] Jonckheere AR (1954). A distribution-free k-sample test against ordered alternatives. Biometrica.

[41] Jonckheere AR (1954). A test of significance for the relation between m rankings and k ranked categories. Br J Stat Psychol.

[42] Ascierto PA, Schadendorf D, Berking C, Agarwala SS, van Herpen CM, Queirolo P, Blank CU, Hauschild A, Beck JT, St-Pierre A, Niazi F, Wandel S, Peters M (2013). MEK162 for patients with advanced melanoma harbouring NRAS or Val600 BRAF mutations: a non-randomised, open-label phase 2 study. Lancet Oncol.

[43] Haigis KM, Kendall KR, Wang Y, Cheung A, Haigis MC, Glickman JN, Niwa-Kawakita M, Sweet-Cordero A, Sebolt-Leopold J, Shannon KM, Settleman J, Giovannini M, Jacks T (2008). Differential effects of oncogenic K-Ras and N-Ras on proliferation, differentiation and tumor progression in the colon. Nat Genet.

[44] Oliveira JB, Bidere N, Niemela JE, Zheng L, Sakai K, Nix CP, Danner RL, Barb J, Munson PJ, Puck JM, Dale J, Straus SE, Fleisher TA (2007). NRAS mutation causes a human autoimmune lymphoproliferative syndrome. Proc Natl Acad Sci USA.

[45] Solit DB, Garraway LA, Pratilas CA, Sawai A, Getz G, Basso A, Ye Q, Lobo JM, She Y, Osman I, Golub TR, Sebolt-Leopold J, Sellers WR (2006). BRAF mutation predicts sensitivity to MEK inhibition. Nature.

[46] Steininger A, Mobs M, Ullmann R, Kochert K, Kreher S, Lamprecht B, Anagnostopoulos I, Hummel M, Richter J, Beyer M, Janz M, Klemke CD, Stein H (2011). Genomic loss of the putative tumor suppressor gene E2A in human lymphoma. J Exp Med.

[47] Goldstein NB, Johannes WU, Gadeliya AV, Green MR, Fujita M, Norris DA, Shellman YG (2009). Active N-Ras and B-Raf inhibit anoikis by downregulating Bim expression in melanocytic cells. J Invest Dermatol.

[48] Kogge A, Volteau C, Saint-Jean M, Peuvrel L, Brocard A, Knol AC, Renaut JJ, Dreno B, Quereux G (2015). Vorinostat for refractory or relapsing epidermotropic T-cell lymphoma: a retrospective cohort study of 15 patients. Acta Derm Venereol.

[49] Zhang J, Chen YL, Ji G, Fang W, Gao Z, Liu Y, Wang J, Ding X, Gao F (2013). Sorafenib inhibits epithelial-mesenchymal transition through an epigenetic-based mechanism in human lung epithelial cells. PLoS One.

[50] Kaltoft K, Bisballe S, Rasmussen HF, Thestrup-Pedersen K, Thomsen K, Sterry W (1987). A continuous T-cell line from a patient with Sezary syndrome. Arch Dermatol Res.

[51] Gootenberg JE, Ruscetti FW, Mier JW, Gazdar A, Gallo RC (1981). Human cutaneous T cell lymphoma and leukemia cell lines produce and respond to T cell growth factor. J Exp Med.

[52] Kaltoft K, Bisballe S, Dyrberg T, Boel E, Rasmussen PB, Thestrup-Pedersen K (1992). Establishment of two continuous T-cell strains from a single plaque of a patient with mycosis fungoides. In Vitro Cell Dev Biol.

[53] Starkebaum G, Loughran TP, Waters CA, Ruscetti FW (1991). Establishment of an IL-2 independent, human T-cell line possessing only the p70 IL-2 receptor. Int J Cancer.

[54] Schmitt M, Pawlita M (2009). High-throughput detection and multiplex identification of cell contaminations. Nucleic Acids Res.

[55] Gulow K, Kaminski M, Darvas K, Suss D, Li-Weber M, Krammer PH (2005). HIV-1 trans-activator of transcription substitutes for oxidative signaling in activation-induced T cell death. J Immunol.

[56] Walczak H, Sprick MR (2001). Biochemistry and function of the DISC. Trends Biochem Sci.

