# Supplementary material for: NRAS mutations in cutaneous T cell lymphoma (CTCL) sensitize tumors towards treatment with the multikinase inhibitor Sorafenib
Source: Oncotarget. 2017 May 7;8(28):45687–97. doi: 10.18632/oncotarget.17669 (PMC5542218; doi:10.18632/oncotarget.17669)
Supplement: Supplementary file 1 [file oncotarget-08-45687-s001.pdf]

NRAS mutations in cutaneous T cell lymphoma (CTCL) sensitize tumors towards treatment with the multikinase inhibitor Sorafenib

Supplementary Materials

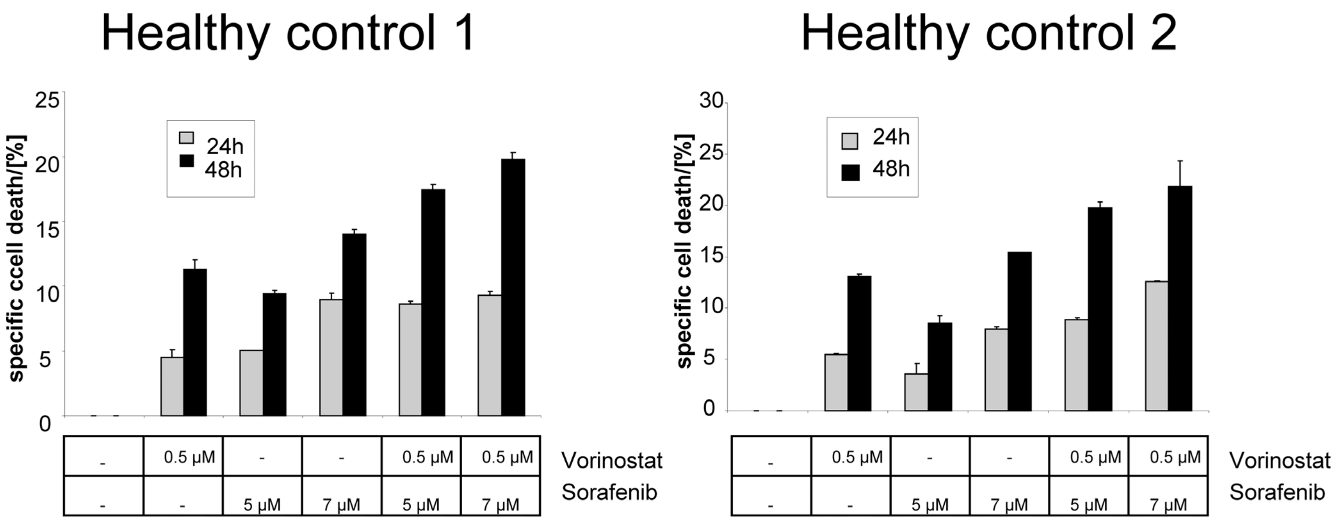

Supplementary Figure 1: Effect of Sorafenib on patients displaying no mutation of RAS compared to healthy controls. (**A**) CD4+ T cells isolated from four different patients were incubated with indicated concentrations of the pan-RAF inhibitor Sorafenib for 48 hours. Then, apoptosis was determined and specific apoptosis was calculated according to the description in materials and methods. (**B**) Same as in A), but CD4+ T cells of healthy controls were used. Specific apoptosis (+/–SD). Shown data is representative.

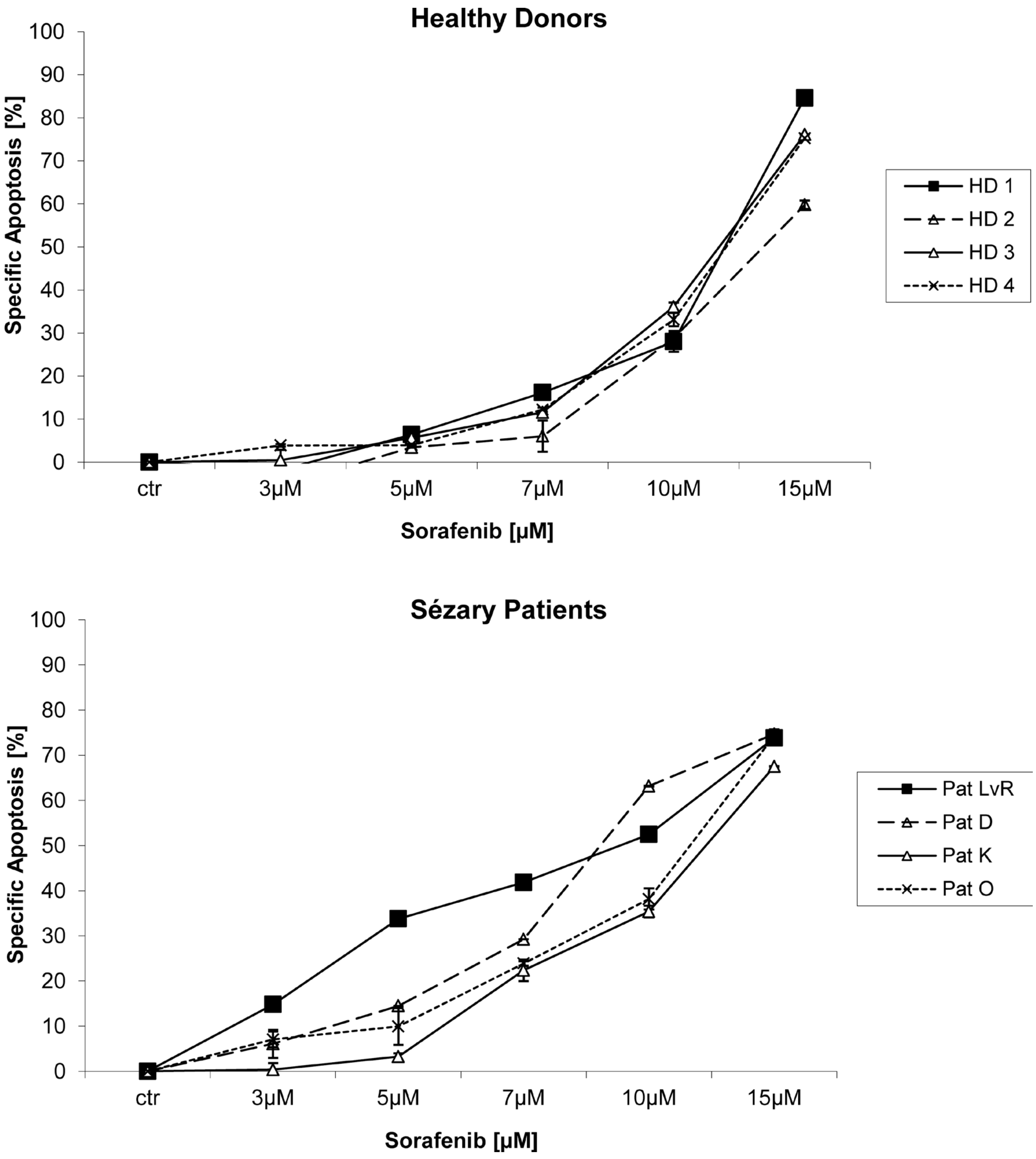

Supplementary Figure 2: Sorafenib and Vorinostat show no cooperative effect in T cells of healthy donors. CD4+ T cells isolated from two different healthy donors were incubated with indicated concentrations of the pan-RAF inhibitor Sorafenib and the HDAC inhibitor Vorinostat for 24 and 48 hours. Then, apoptosis was determined and specific apoptosis was calculated according to the description in materials and methods.

Supplementary Table 1: Patient information (f: female; TNM: T = tumor size, *N* = (regional) lymph node involvement, M = (distant) metastasis)

Patient	gender, age	TNM	Leukocyte/nl	T lymphocytes	CD4/µl	Sezary cell count/µl	CD4/CD8	skin involvement	% skin involved
LvR	f, 64	T4N0M0B1	10,67	92.7%	3985	3851	11,8	severe	100
K	f, 56	T4N0M0B1	13,69	96.0%	4160	1488	19,0	severe	100
O	f, 61	T4N0M0B1	5,17	48.0%	179	37	1,7	severe	90
D	f, 67	T4N0M0B1	9,51	92.7%	2131	1870	28,2	severe	80
Norm			4,2–10,2	60–83%	528–1495	< 1000	1–2,8		
